# Improving dose delivery in non‐coplanar cranial SRS: Stereoscopic x‐ray‐guided mitigation of table walkout errors

**DOI:** 10.1002/acm2.70099

**Published:** 2025-04-09

**Authors:** Yohan A. Walter, Philip F. Durham, Anne N. Hubbard, William E. Burrell, Hsinshun T. Wu

**Affiliations:** ^1^ Department of Radiation Oncology Willis Knighton Cancer Center Shreveport Louisiana USA; ^2^ Department of Clinical Research University of Jamestown Fargo North Dakota USA

**Keywords:** linear accelerator, QA, SRS, stereotactic radiation therapy, stereotactic radiosurgery

## Abstract

**Purpose:**

Linear accelerator (LINAC)‐based single‐isocenter multi‐target (SIMT) treatment has streamlined the cranial stereotactic radiosurgery (SRS) workflow. Though efficient, SIMT delivery adds additional challenges that should be considered, including increased sensitivity to rotational errors for off‐isocenter targets. Room‐mounted imaging systems carry the advantage of allowing fast, low‐dose imaging at nonzero couch angles, which may combat the effects of table walkout and residual rotational errors. Here, we performed a series of end‐to‐end tests to determine if these corrections correlate with a measurable difference in delivered dose and to assess the overall accuracy of SIMT delivery on our LINAC‐based SRS platform.

**Methods:**

Ten treatment plans of increasing complexity were created in the Elements 4.0 treatment planning system (TPS, Brainlab AG). Plans were delivered on an Elekta Versa HD LINAC (Elekta AB) with the ExacTrac (ETX) imaging system (Brainlab AG). A CT scan of a StereoPHAN with SRS MapCHECK (Sun Nuclear) was imported into the TPS. Measured targets were contoured on the detector plane. Plans used 4–15 treatment arcs and 4–6 couch angles. ETX was used for initial phantom positioning. Dose measurements were performed for each plan with and without ETX‐guided corrections at all table angles.

**Results:**

Translational and rotational residual shifts were all submillimeter and ≤1.0 degrees, respectively, across all table angles. Using 3.0%/1.0 mm gamma criteria, all gamma pass rates (GPR) were either equal or improved when ETX shifts were executed, though the difference was not statistically significant (*p* = 0.076). However, using 2.0%/0.5 mm criteria, GPR improved significantly (*p* = 0.016) with ETX repositioning. The average GPR improvement was 4.5% ± 4.8%.

**Conclusions:**

Results demonstrate that repositioning corrections at each table angle improve agreement between planned and delivered dose at the submillimeter level. The test treatment plans in this study may be used for assessment of end‐to‐end treatment delivery accuracy for complex LINAC‐based stereotactic radiotherapy procedures.

## 1 INTRODUCTION

Linear accelerator (LINAC)‐based single‐isocenter multi‐target (SIMT) treatment has streamlined the cranial stereotactic radiosurgery (SRS) workflow. However, off‐isocenter treatment carries multiple additional difficulties compared to isocentric treatment, including increased sensitivity to rotational errors,^1^, ^2^, ^3^, ^4^ which may result in significant discrepancies between planned and delivered dose.

Sources of rotational error include initial patient positioning, intrafraction patient motion, and mechanical uncertainties.^3^, ^5^, ^6^ A recent longitudinal study identified table walkout as the most significant factor resulting in a loss of mechanical isocentricity over a 5‐year period.^7^ The study noted that precise adjustments to the couch isocenter can be difficult to perform, and walkout‐related uncertainties still persisted despite routine adjustment.^7^

Since on‐board cone beam computed tomography (CBCT)‐based image guidance is only possible at small table angles, both translational and rotational errors at nonzero couch angles can go undetected and may impact overall alignment in noncoplanar treatment delivery. Various methods have arisen which may reduce these potential errors, including surface guidance systems and x‐ray‐based methods capable of imaging at nonzero table angles.^8^, ^9^, ^10^, ^11^ Image‐guided table shifts using these systems may therefore correct for couch‐and patient motion‐related positional uncertainties that may otherwise go uncorrected in a workflow using solely CBCT‐based alignment.

Theoretically, the agreement between planned and delivered dose should improve with corrections performed at each table angle.^2^, ^12^ To our knowledge, however, the effects of correcting couch walkout on delivered dose have not been directly measured for SIMT SRS treatments outside of treatment planning system (TPS)‐based studies.^2^ Though the computational studies may model dosimetric impacts of table walkout, it is crucial to evaluate effects in the context of treatment delivery to determine end‐to‐end effects. Considering that the correction methods, namely, table movement and image registration, have their own associated uncertainties, computational modeling may not adequately account for all sources of error present in treatment delivery.

In this study, we developed a series of end‐to‐end treatment plans of increasing complexity which simulated a range of clinical scenarios. We then performed dose measurements to determine if couch positional corrections performed during treatment delivery correlate with a measurable difference in the delivered dose. In addition to elucidating the role of image guidance for noncoplanar SRS treatment, the methods used herein may serve as a template for evaluation of other SRS platforms.

## 2 METHODS

### 2.1 End‐to‐end test plans

Ten treatment plans of increasing complexity were developed, influenced by the TG‐119 methodology.^13^ A summary of the plan descriptions is listed in Table 1. Further plan details are provided in the supplementary material (Figures S1–S10, Tables S1, S2).

**TABLE 1:** Summary of test cases.

Plan #	# Targets on detector plane	# Table angles	# Arcs	DCA or hybrid?	Summary
1	1	5	5	DCA	Single target at isocenter (Figure S1)
2	1	5	10	DCA	Single target off‐isocenter (Figure S2)
3	2	5	9	DCA	2 small targets off‐isocenter (Figure S3)
4	4	4	8	DCA	4 small targets on‐plane (Figure S4)
5	4	5	10	DCA	4 small targets off‐plane (Figure S5)
6	2	5	8	Hybrid	Large + small target on‐plane (Figure S6)
7	1	5	13	Hybrid	Large target off‐isocenter (Figure S7)
8	2	5	8	Hybrid	C‐shape + small target on‐plane (Figure S8)
9	2	5	13	Hybrid	C‐shape + small target off‐isocenter (Figure S9)
10	6	6	15	Hybrid	C‐shape + 5 targets, one overlapping C‐shape (Figure S10)

*Note*: Targets on the detector plane were measured. Note that supplementary dummy targets were used to shift the isocenter away from the detector plane. Refer to Figures S1‐S10 in the supplementary material for images of each plan.

Abbreviations: DCA, dynamic conformal arcs; Hybrid, hybrid DCA‐VMAT optimization.

The StereoPHAN with an inserted SRS MapCHECK (Sun Nuclear, Melbourne, FL, USA) was used for end‐to‐end testing. The SRS MapCHECK features 1013 diode detectors, each with a 0.48 mm active volume, spaced 2.47 mm apart on a single plane. Based on the manufacturer's recommendations, a 1.20 g/cc density override was applied to the phantom in the TPS for treatment planning.

A scan of the phantom was taken with 1.0 mm slice thickness on a Philips Big Bore RT CT simulator (Philips, Andover, MA, USA). Images were imported into the Brainlab Elements version 4.0 TPS (Brainlab AG, Munich, Germany). Contours of mock organs at risk, including the brainstem, optic pathway, eyes, cochleae, and brain, were drawn on the phantom scan to simulate a clinically realistic treatment geometry. Targets were drawn on the SRS MapCHECK detector plane for measurement. As needed, the plan isocenter was shifted by adding mock targets off‐plane. Eight distinct targets were drawn with characteristics described in Table 2.

**TABLE 2:** Summary of target characteristics.

Target name	Volume (cc)	Target maximum diameter (cm)	Description
PTV 01	1.91	1.80	Mid‐sized target on detector plane. Target overlaps “C‐shape” (PTV 08) in plan 10 (Figure S10).
PTV 02	0.25	0.92	Small targets drawn on the four corners of detector plane (PTV 02–05).
PTV 03	0.24	0.85	
PTV 04	0.27	1.01	
PTV 05	0.24	0.83	
PTV 06	35.60	5.24	Large target centered on detector plane.
PTV 07	0.17	0.72	Very small target on detector plane.
PTV 08	12.35	4.51	“C‐shape” with spherical avoidance structure in its center.

*Note*: All listed targets were contoured on the detector plane. Supplement dummy targets were drawn off‐plane to shift isocenter away from the SRS MapCHECK detector plane.

Abbreviation: PTV, planning target volume.

Treatment plans were developed using our clinical dose goals. The arc arrangements and dose goals are listed in the supplementary material (Tables S1, S2). Plans were designed to deliver the 35 Gy prescription dose to 98% of each planning target volume (PTV). The treatment isocenter was placed automatically by the Brainlab system at the geometric center of the targets, except for case 8, where the isocenter was placed at the center of the C‐shaped target. In cases 8–10, which all used the C‐shaped target, a spherical avoidance structure was placed in the opening of the “C” to influence plan complexity and dose shaping.

All plans used a 6 MV flattening filter free (FFF) beam, four to six distinct table angles, and 4 to 15 gantry arcs, delivered on an Elekta Versa HD (Elekta AB, Stockholm, Sweden) linear accelerator with 5.0 mm leaf width projected at isocenter. All table angles between 270 degree and 90 degree positions (IEC 61217 coordinates) in 10‐degree increments were used in at least one treatment plan.

Both dynamic conformal arc (DCA)‐only and hybrid optimization were used. Hybrid optimization, available in the Elements 4.0 TPS, allows users to select one metastasis to be treated with three distinct arcs using volumetric modulated arc therapy (VMAT), while the remaining targets are treated with DCA.

Plan doses were calculated using our clinical reference beam model‐based Monte Carlo dose calculation algorithm with 1.0 mm grid resolution and 2.0% calculation uncertainty, reporting dose to medium.

### 2.2 Dose measurement

Prior to each dose measurement, an off‐isocenter Winston–Lutz test was performed to verify mechanical alignment within 1.0 mm for tested combinations of gantry, couch, and collimator rotations using two targets: one at isocenter, and one 5.6 cm from isocenter. The tested fields and day‐of‐measurement Winston–Lutz results are summarized in the supplementary material (Tables S3, S4). The ExacTrac (ETX) kilovoltage planar oblique x‐ray system (Brainlab AG, Munich, Germany) was used for initial phantom setup, but no further corrections were made at tested table angles.

**FIGURE 1:**
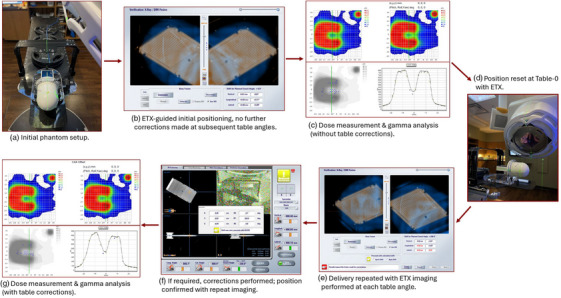
Dose measurement workflow. (a) The phantom was aligned with the treatment plan isocenter. 180‐lb (81 kg) of weight was placed on the table. (b) ExacTrac (ETX) image registration was used for precise phantom alignment at table‐0 degrees. (c) the plan was delivered without further image‐guided positional corrections and gamma analysis was performed. (d) the phantom position was reset at table‐0 degrees using ETX image guidance. (e) the same treatment plan as used in step A‐C was delivered with ETX images taken at each table angle. (f) if repositioning shifts exceeded 0.4 mm or 0.4 degrees, repositioning shifts were executed before delivery of arcs at each table angle. (g) gamma analysis was performed.

The dose measurement workflow is outlined in Figure 1. Following plan generation, treatment plans were exported to the MOSAIQ record and verify (R&V) system (Elekta AB, Stockholm, Sweden). Reference image data was exported to the ETX system.

The StereoPHAN with SRS MapCHECK was placed on the treatment table. 180 lb (81 kg) of additional weight was stacked on the couch to simulate patient load (Figure 1a). The 6‐degree‐of‐freedom (DoF) HexaPOD couch was used for initial phantom positioning (Figure 1b). Shifts were acquired using ETX with automatic image registration. All registrations were verified by a physicist prior to executing shifts. The thresholds for repositioning were 0.4 mm and 0.4 degrees. If required shifts exceeded either threshold, repositioning was performed using the HexaPOD system, and the final table position was confirmed with repeat imaging.

Following initial image‐guided phantom positioning, dose measurements were taken for delivery without (Figure 1c) and with further ETX‐guided 6‐DoF corrections at every table angle (Figure 1e,f). Measurements with and without corrections were taken back‐to‐back to eliminate daily mechanical and dosimetric variations (Figure 1d). Dose distributions measured using the SRS MapCHECK were compared against the treatment plan using gamma analysis. 2.0% and 3.0% dose difference (DD) with 0.5 and 1.0 mm respective distance‐to‐agreement (DTA) gamma criteria and 10% threshold were used for comparison. Additionally, the maximum dose in each measured target on the detector plane was compared against the TPS calculation to give the peak dose difference (PD).

### 2.3 Statistical analysis

An unpaired *t*‐test was used to compare the individual directional and rotational components of table walkout. Paired *t*‐tests were used to determine the effect of table corrections on gamma pass rates (GPR) and measured PD. Statistical significance was defined by *p* < 0.05. All analyses were performed in STATA version 18.0 (StataCorp LLC, College Station, TX, USA).

## 3 RESULTS

### 3.1 Table walkout and ETX repositioning shifts

**FIGURE 2:**
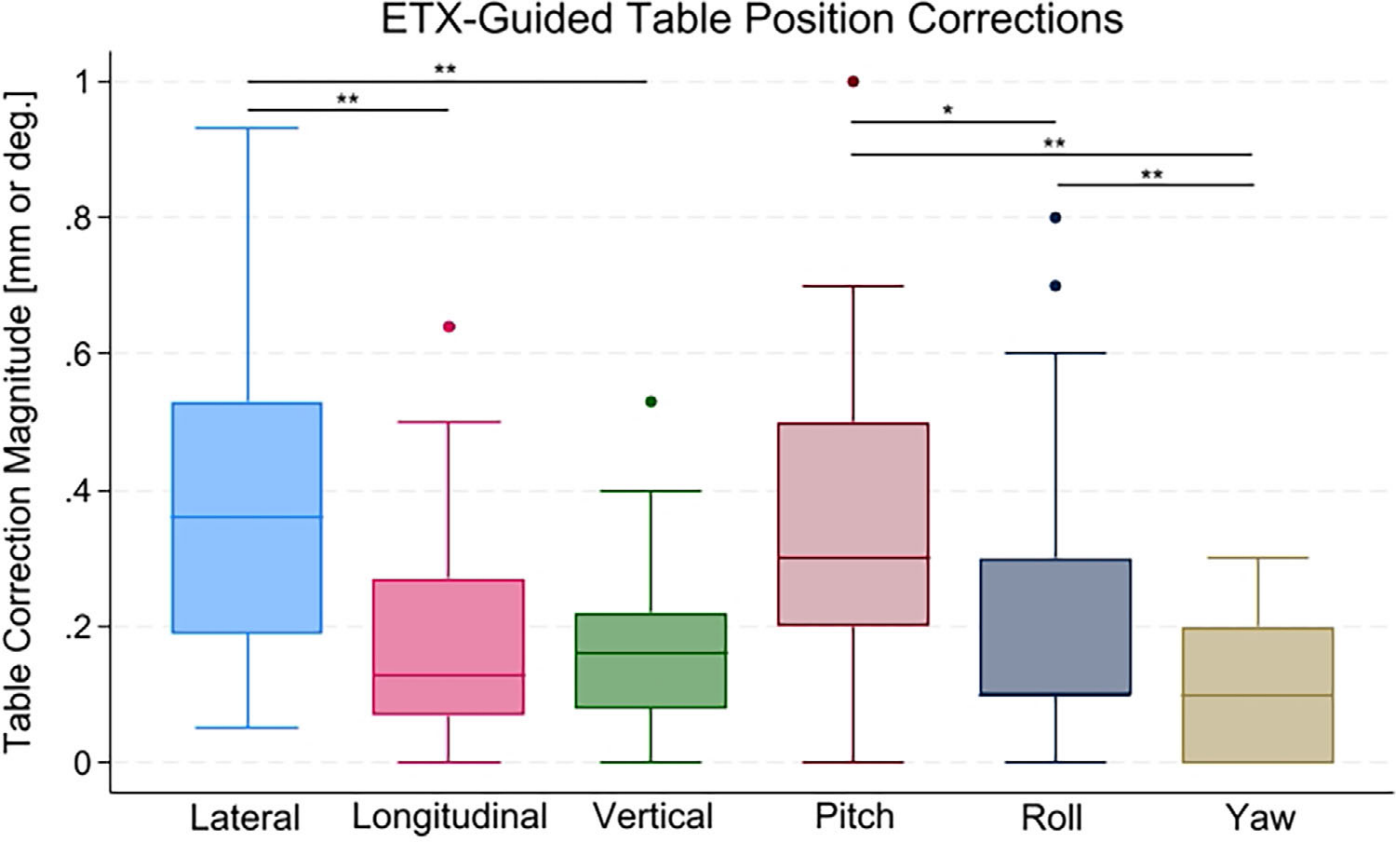
ExacTrac (ETX)‐guided table corrections at nonzero table angles for all six degrees of freedom. * indicates *p* < 0.05, ** indicates *p* < 0.01.

Based on the image registration during treatment delivery, the maximum translational and rotational shifts were 0.93 mm and 1.0 degrees, respectively. The average translational and rotational walkout measured for each plan are listed in Table 3. Across all table angles, the average magnitude of lateral, longitudinal, and vertical translations were 0.38 ± 0.22 mm, 0.18 ± 0.16 mm, and 0.17 ± 0.13 mm, respectively. The average residual pitch, roll, and yaw were 0.31 ± 0.22 degrees, 0.22 ± 0.19 degrees, and 0.12 ± 0.11 degrees, respectively. Translational and rotational walkout were most significant in the lateral direction (*p* < 0.01) and pitch (*p* < 0.05), respectively (Figure 2).

**TABLE 3:** Summary of measurement results.

Plan #	Avg. translational walkout (mm)	Avg. rotational walkout (degrees)	GPR (%), 3%/1.0 mm, ETX corrected	GPR (%), 3%/1.0 m m, without ETX correction	GPR (%), 2%/0.5 mm, ETX corrected	GPR (%), 2%/0.5 mm, without ETX correction	Target	Dist. from iso (mm)	Peak dose difference (%), ETX corrected	Peak dose difference (%), without ETX correction
1	0.19	0.23	100.0	100.0	93.6	93.3	PTV 01	0	1.20	1.32
2	0.31	0.18	99.4	99.4	84.1	88.7	PTV 01	51	−1.53	−1.54
3	0.26	0.15	99.5	87.9	75.7	63.1	PTV 02	50	0.37	0.93
							PTV 03	75	0.28	0.46
4	0.33	0.41	99.5	98.0	85.3	84.1	PTV 04	45	0.24	0.14
							PTV 05	45	−0.42	−2.13
							PTV 02	45	−0.94	−0.46
							PTV 03	45	−0.98	−0.83
5	0.23	0.15	100.0	99.2	81.7	79.2	PTV 04	39	−1.13	−0.91
							PTV 05	25	−0.94	0.83
							PTV 02	61	−0.43	0.18
							PTV 03	69	−0.12	−0.04
6	0.22	0.28	99.5	98.9	85.0	79.0	PTV 06 (V)	22	−0.91	−1.02
							PTV 07	22	−1.61	−1.69
7	0.16	0.15	99.5	99.1	92.0	83.9	PTV 06 (V)	42	−0.37	−0.78
8	0.29	0.26	98.4	95.8	87.3	81.7	PTV 08 (V)	0	−0.21	−0.41
							PTV 05	62	1.46	−4.44
9	0.32	0.24	99.0	96.7	91.1	83.6	PTV 08 (V)	60	−0.09	−0.32
							PTV 05	7	0.06	0.19
10	0.21	0.20	95.7	93.7	79.9	73.8	PTV 08 (V)	15	−3.12	−3.59
							PTV 04	43	−2.10	−0.96
							PTV 05	50	−0.55	0.77
							PTV 02	46	−4.03	−5.13
							PTV 03	40	−3.40	−3.86
							PTV 01	12	−0.30	−0.14

*Note*: Metrics for measurements run with ETX corrections at each table angle are listed. Results for the measurements without ETX corrections are indicated in parentheses. (V) indicates a target treated with VMAT. All others were treated with DCA.

Abbreviations: ETX, ExacTrac; GPR, gamma pass rate; PTV, planning target volume.

Repositioning shifts were executed when either translational or rotational corrections exceeded 0.4 mm or 0.4 degrees, respectively. Repositioning was performed for 28 of the 41 nonzero table positions across all ten plans (68.3%). All 10 plans required ETX correction for at least one table angle, though no visible patterns emerged which correlated required shifts with specific table angles.

### 3.2 Gamma analysis

Using the 3.0% DD and 1.0 mm DTA criteria, all GPR were either equivalent to or improved when ETX corrections were performed as compared to the GPR without corrections (Table 3). When ETX corrections were performed, GPRs for all 10 plans were above our clinically acceptable 95.0% minimum. Without corrections, cases 3 and 10 had GPRs below the acceptable threshold (87.9% and 93.7%, respectively). On average, gamma pass rates improved by 2.2% ± 3.4%, though the difference was not statistically significant (*p* = 0.076). Gamma pass rates with and without ETX corrections averaged 99.1% ± 1.3% and 96.9% ± 3.7%, respectively.

**FIGURE 3:**
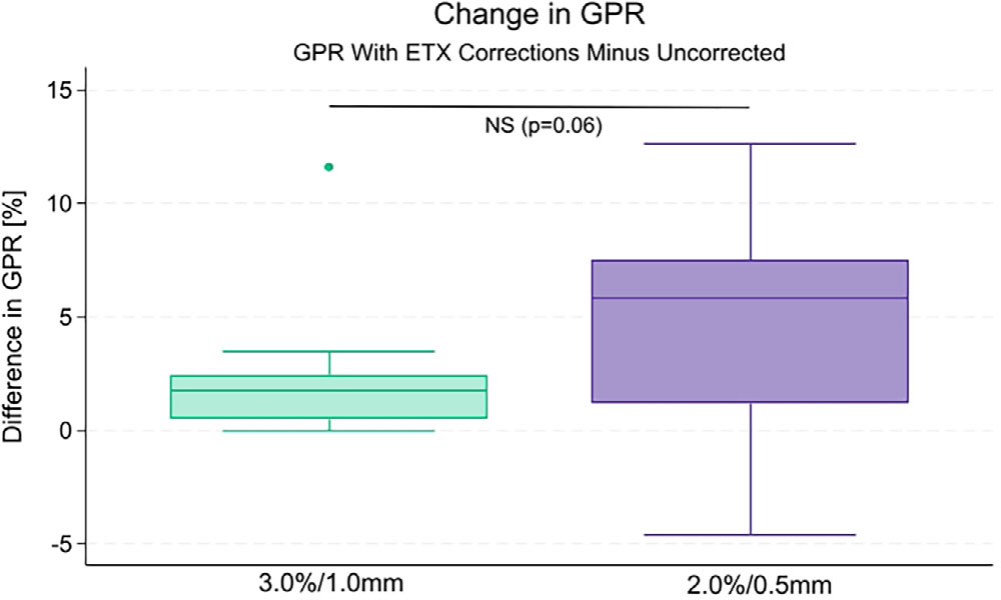
Changes in the gamma pass rate (GPR) between plans delivered with and without ExacTrac (ETX)‐guided table corrections for the 3.0%/1.0 mm and 2.0%/0.5 mm dose difference/distance‐to‐agreement criteria.

At the 2.0% DD and 0.5 mm DTA level, similar trends arose (Figure 3). All but one case (case 2) showed an improvement in GPR when using ETX corrections. The difference between ETX‐corrected and non‐corrected GPR was statistically significant (*p* = 0.016). Gamma pass rates with and without ETX corrections averaged 85.6% ± 5.6% and 81.0% ± 8.3%, respectively.

### 3.3 Peak dose difference

Differences in PD between ETX‐corrected and non‐corrected measured doses were variable across the ten cases. The most dramatic difference in peak dose was observed for case 8, where PTV 05 had a PD of 1.46% with ETX corrections, and −4.44% without corrections, relative to the TPS‐predicted dose. The magnitude of discrepancy between measured and TPS maximum dose was 1.19% ± 1.24% with corrections, and 1.32% ± 1.42% without ETX corrections, averaged across all measured targets, marking a marginal, but not statistically significant impact of ETX corrections on peak dose agreement (*p* = 0.78).

## 4 DISCUSSION

A study by Brezovich et al. found that, despite measuring table walkout up to 0.6 mm in magnitude, the resultant shift in the dose cloud at isocenter was much smaller (0.16 mm), as determined via TPS modeling,^12^ suggesting that table walkout which, on the surface, may seem significant, could translate to small, nearly negligible, shifts in the dose cloud. Though other studies have modeled the effects of table walkout using phantoms or TPS calculations,^1^, ^2^, ^12^ these models may underestimate the effect of walkout‐induced translational and rotational errors on off‐isocenter targets, as they may neglect some key sources of error in treatment delivery, such as image registration and table movement uncertainties. Considering the widespread use of noncoplanar single‐isocenter, multi‐target linear accelerator‐based SRS, deeper characterization of the effect on delivered dose can further inform future protocols and assist users in selecting SRS platforms.

In a workflow that mirrors patient treatment, ETX image registrations performed during plan delivery demonstrated good mechanical alignment, with all residual table translations under 1.0 mm for all 41 nonzero table angles tested, and all residual table rotations ≤1.0 degrees. However, there were multiple instances where the pitch and roll corrections exceeded 0.5 degrees, highlighting the importance of considering rotational effects of table walkout in SIMT delivery.

Despite only 28 of the 41 positions exceeding our thresholds for repositioning, there was an observable difference in the gamma pass rates between the table‐corrected and non‐corrected dose distributions; however, when using the clinical 3.0%, 1.0 mm gamma criteria, the difference was not statistically significant. At the 2.0%, 0.5 mm level, the difference was significant (*p* = 0.016), which suggests that the net effects of table walkout and residual rotational errors are significant shifts in dose clouds at the submillimeter level.

Additionally, the marginal improvement in peak dose discrepancies between measured and calculated dose suggest that the positional errors most directly caused shifts in the dose cloud position, rather than changes to the dose level within the target. However, precise determination of the peak dose may be limited in part by the resolution of the SRS MapCHECK array, where diodes were spaced 2.47 mm apart and had a 0.48 mm active volume. Though the detector resolution is sufficient for SRS patient‐specific quality assurance, repeated measurements of peak dose differences with higher‐resolution systems may yield different results.

Though our findings generally agree with previous reports which suggested a small impact of table walkout on delivered dose,^12^ it is crucial to note that, with ETX corrections, gamma pass rates nearly unanimously improved, indicating better agreement between the delivered and planned dose. Though small, mitigating these positional errors measurably improves delivery accuracy. Since positional errors such as those highlighted in this study factor into the uncertainties limiting end‐to‐end delivery accuracy, minimizing these discrepancies can further facilitate linear accelerator‐based radiosurgery with submillimeter precision, thereby potentially reducing the necessary PTV margin expansion. Paired with a formal assessment of intrafraction patient motion and other mechanical uncertainties, these findings may support reassessment of PTV margin requirements for linear accelerator‐based SRS using noncoplanar arcs.

A limitation of this study is that results of similar testing will vary for individual systems and machine designs. However, our machine adheres to the mechanical performance tolerances described in TG‐142 and MPPG 9a.^14^, ^15^ Therefore, other users following similar guidelines may see similar results, and the data reported here may be representative of well‐maintained linear accelerators used for SRS.

## 5 CONCLUSION

The results of this study highlight the need for effective and rigorous quality assurance in ensuring minimal effects of table walkout. Though the effects of table walkout on dose distributions were small, the impact on delivered dose was measurable at the submillimeter level. Furthermore, in view of the sensitivity of SIMT treatment to rotational errors, drift in the pitch and roll, as well as residual yaw corrections must be considered. However, these errors may only be detected by off‐isocenter Winston–Lutz tests, or walkout investigations similar to those performed in this study. Results highlight the value in image guidance systems capable of performing positional corrections at nonzero table angles. Users performing SIMT SRS procedures without fiducial, surface, or x‐ray‐based tracking may need to perform rigorous routine evaluations of table walkout to ensure acceptable mechanical performance.

## CONFLICT OF INTEREST STATEMENT

YAW and HTW disclose active research agreement with CQ Medical (Avondale, PA, USA). YAW reports receiving travel and meeting registration funding from CQ Medical. WEB reports consulting work for C‐RAD AB (Stockholm, Sweden).

## Supporting information



Supporting Information

## Data Availability

The data that support the findings of this study are available from the corresponding author upon reasonable request.
