# Supplementary material for: Improving dose delivery in non‐coplanar cranial SRS: Stereoscopic x‐ray‐guided mitigation of table walkout errors
Source: J Appl Clin Med Phys. 2025 Apr 9;26(6):e70099. doi: 10.1002/acm2.70099 (PMC12148762; doi:10.1002/acm2.70099)
Supplement: Supplementary file 1 — Supporting Information [file ACM2-26-e70099-s001.docx]

**Supplementary Material:** Improving Dose Delivery in Non-Coplanar Cranial SRS: Stereoscopic X-Ray-Guided Mitigation of Table Walkout Errors

**Summary of cases:**

Figures S1-S10 are screenshots of coronal and sagittal views of each test plan used in this study. Please refer to Table 1 for descriptions of the test plans and Table 2 for the descriptions of individual targets.


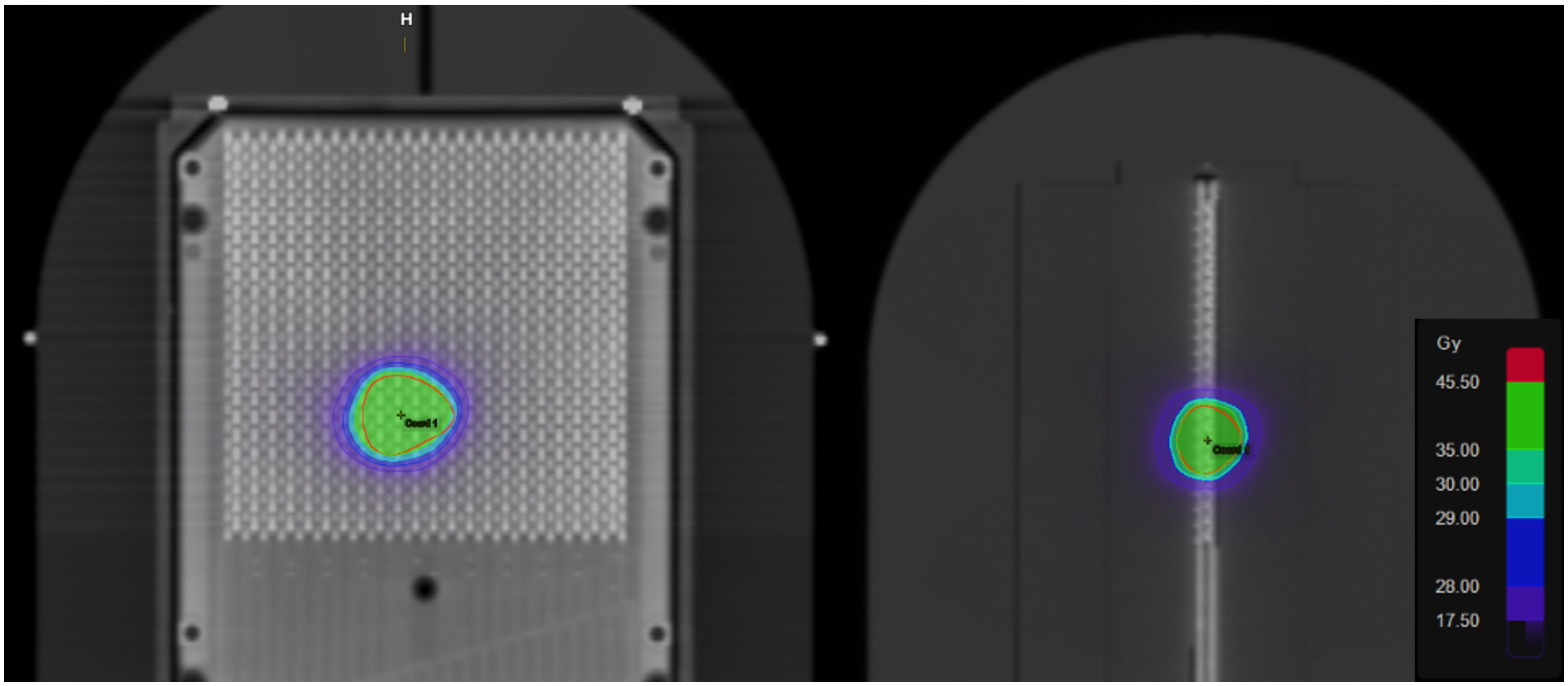


Figure S1: Coronal (left) and sagittal (right) view of case 1 targets and dose distributions. Targets are indicated by solid lines; dose is indicated by the color wash. Plan isocenter is indicated by the crosshairs.


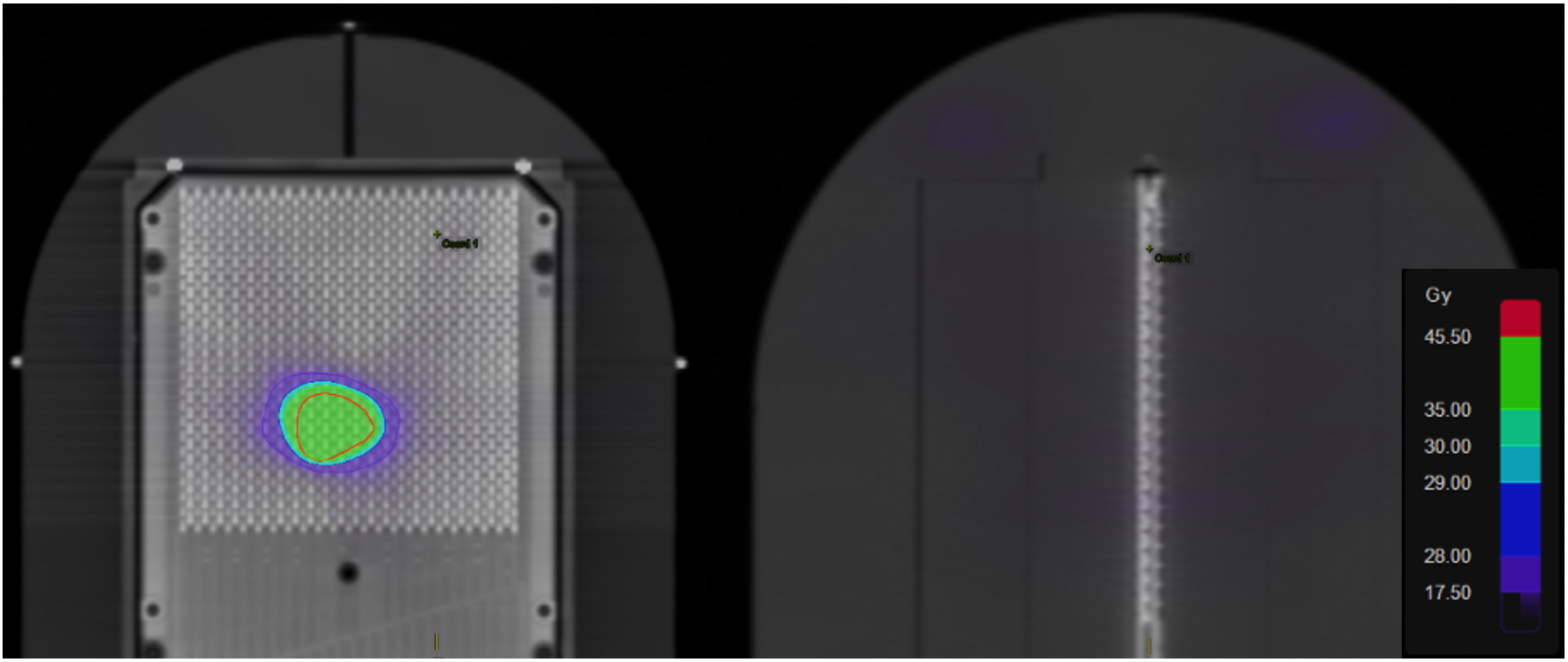


Figure S2: Coronal (left) and sagittal (right) view of case 2 targets and dose distributions. Targets are indicated by solid lines; dose is indicated by the color wash. Plan isocenter is indicated by the crosshairs.


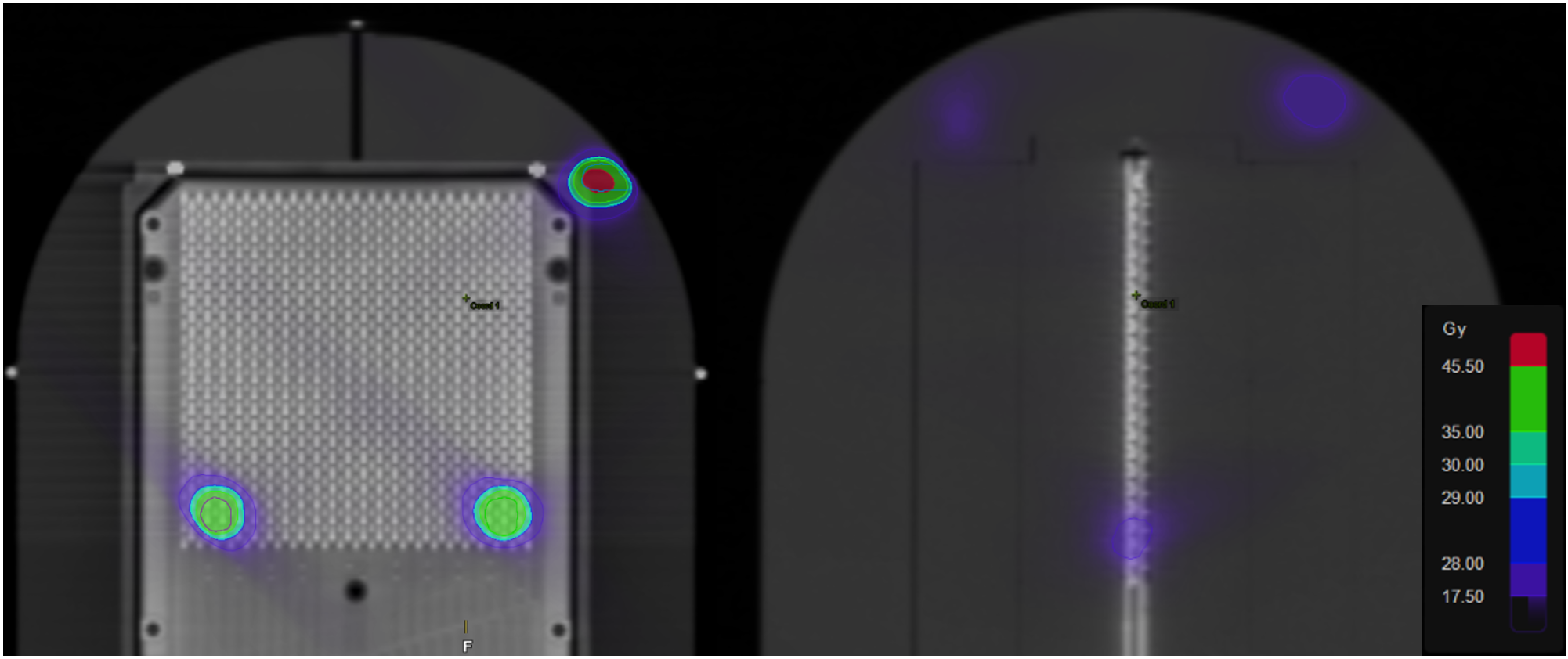


Figure S3: Coronal (left) and sagittal (right) view of case 3 targets and dose distributions. Targets are indicated by solid lines; dose is indicated by the color wash. Plan isocenter is indicated by the crosshairs. Note that targets drawn off the detector plane were used to shift the treatment isocenter.


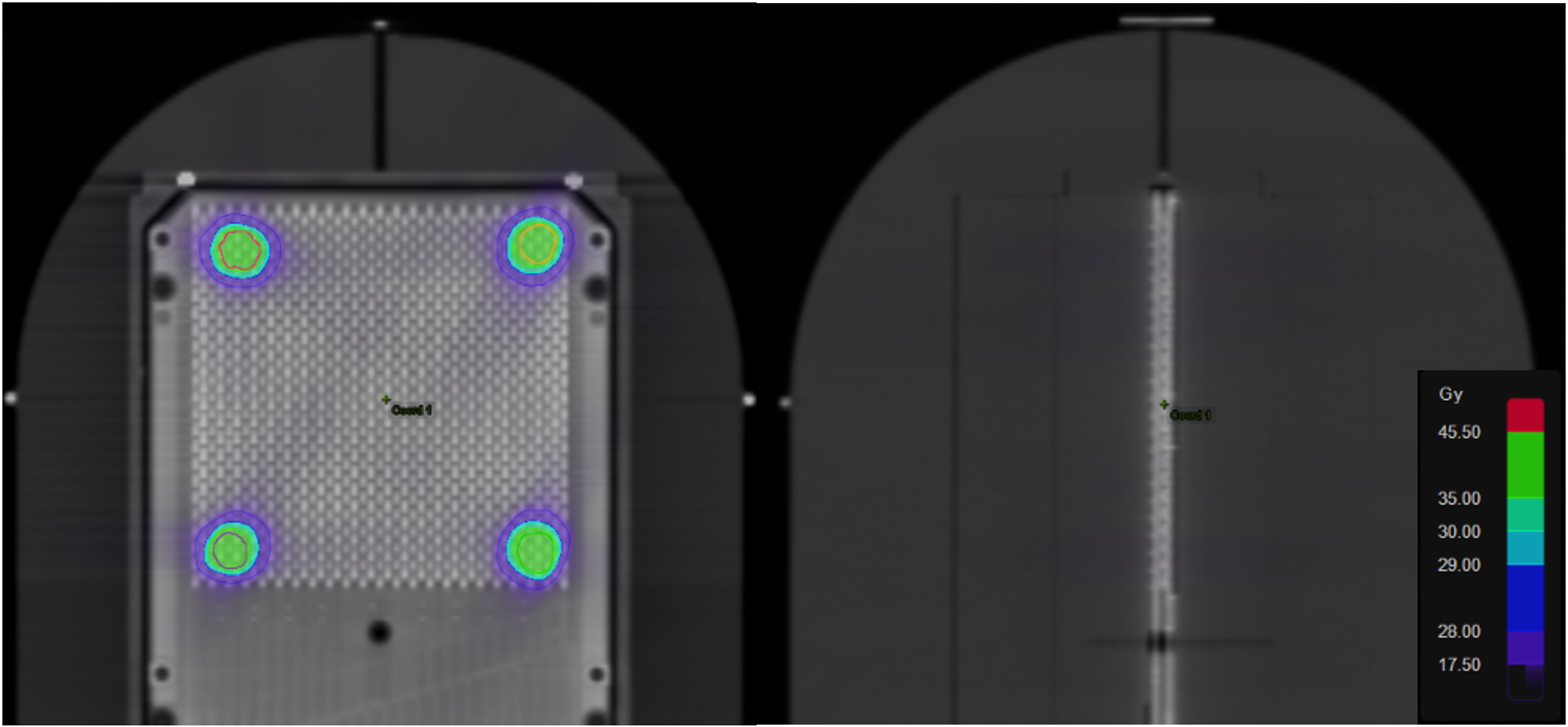


Figure S4: Coronal (left) and sagittal (right) view of case 4 targets and dose distributions. Targets are indicated by solid lines; dose is indicated by the color wash. Plan isocenter is indicated by the crosshairs.


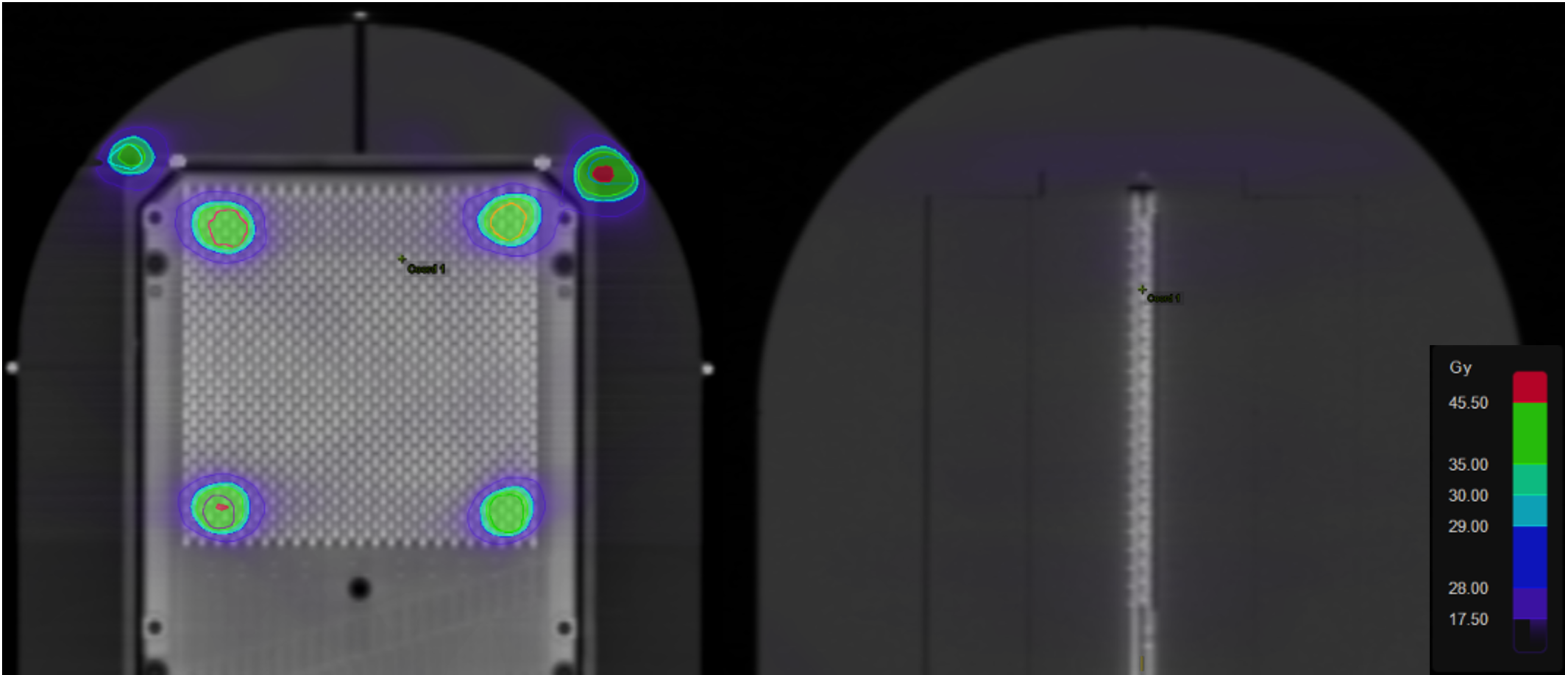


Figure S5: Coronal (left) and sagittal (right) view of case 5 targets and dose distributions. Targets are indicated by solid lines; dose is indicated by the color wash. Plan isocenter is indicated by the crosshairs. Note that targets drawn off the detector plane were used to shift the treatment isocenter.


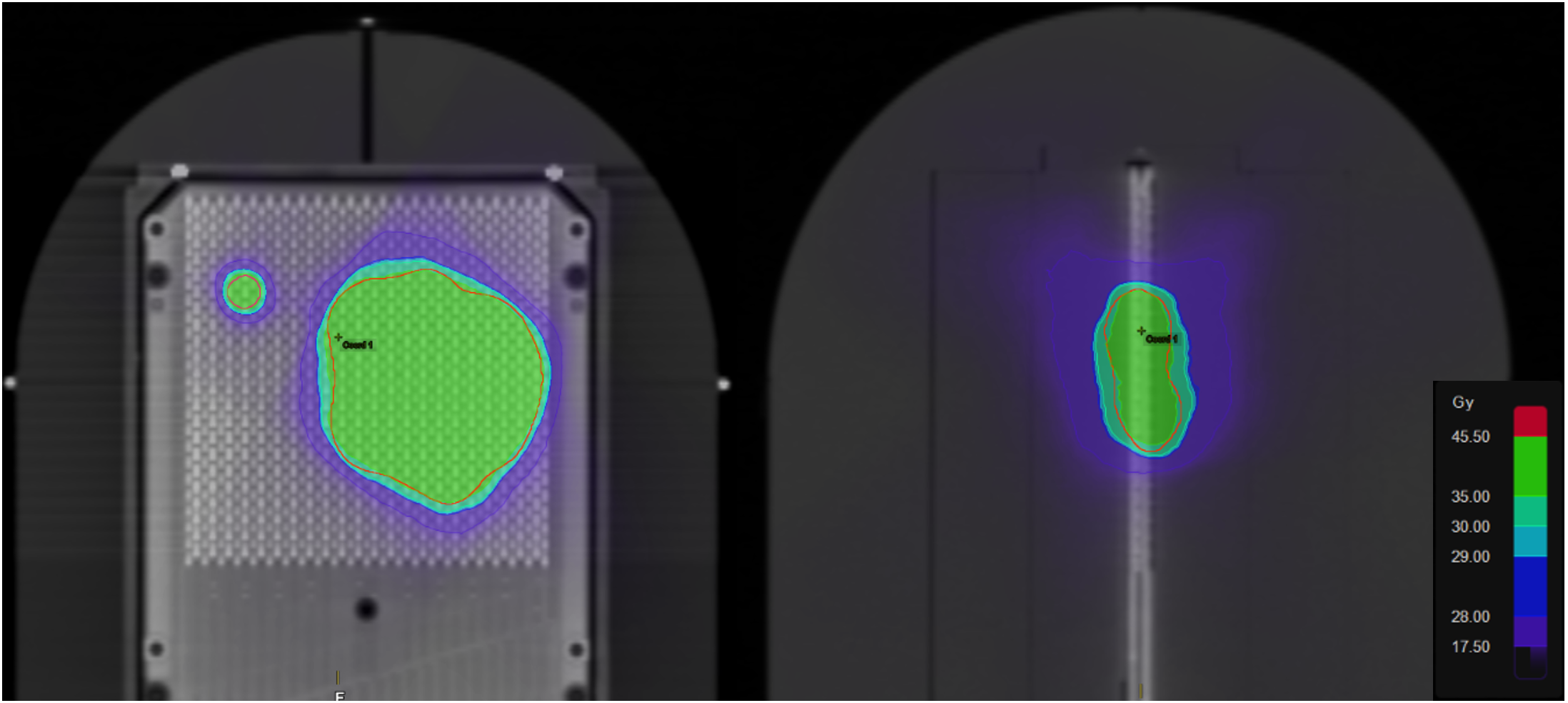


Figure S6: Coronal (left) and sagittal (right) view of case 6 targets and dose distributions. Targets are indicated by solid lines; dose is indicated by the color wash. Plan isocenter is indicated by the crosshairs.


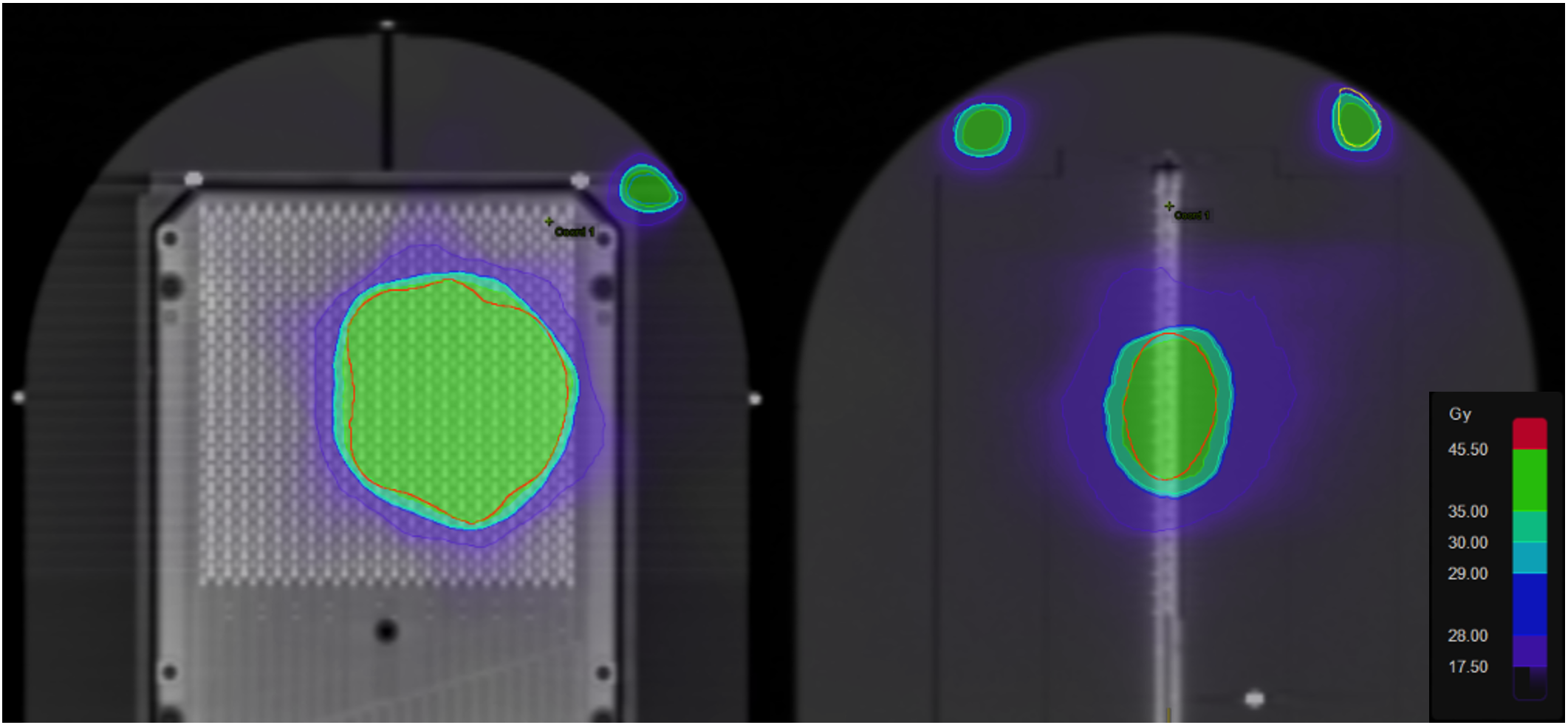


Figure S7: Coronal (left) and sagittal (right) view of case 7 targets and dose distributions. Targets are indicated by solid lines; dose is indicated by the color wash. Plan isocenter is indicated by the crosshairs. Note that targets drawn off the detector plane were used to shift the treatment isocenter.


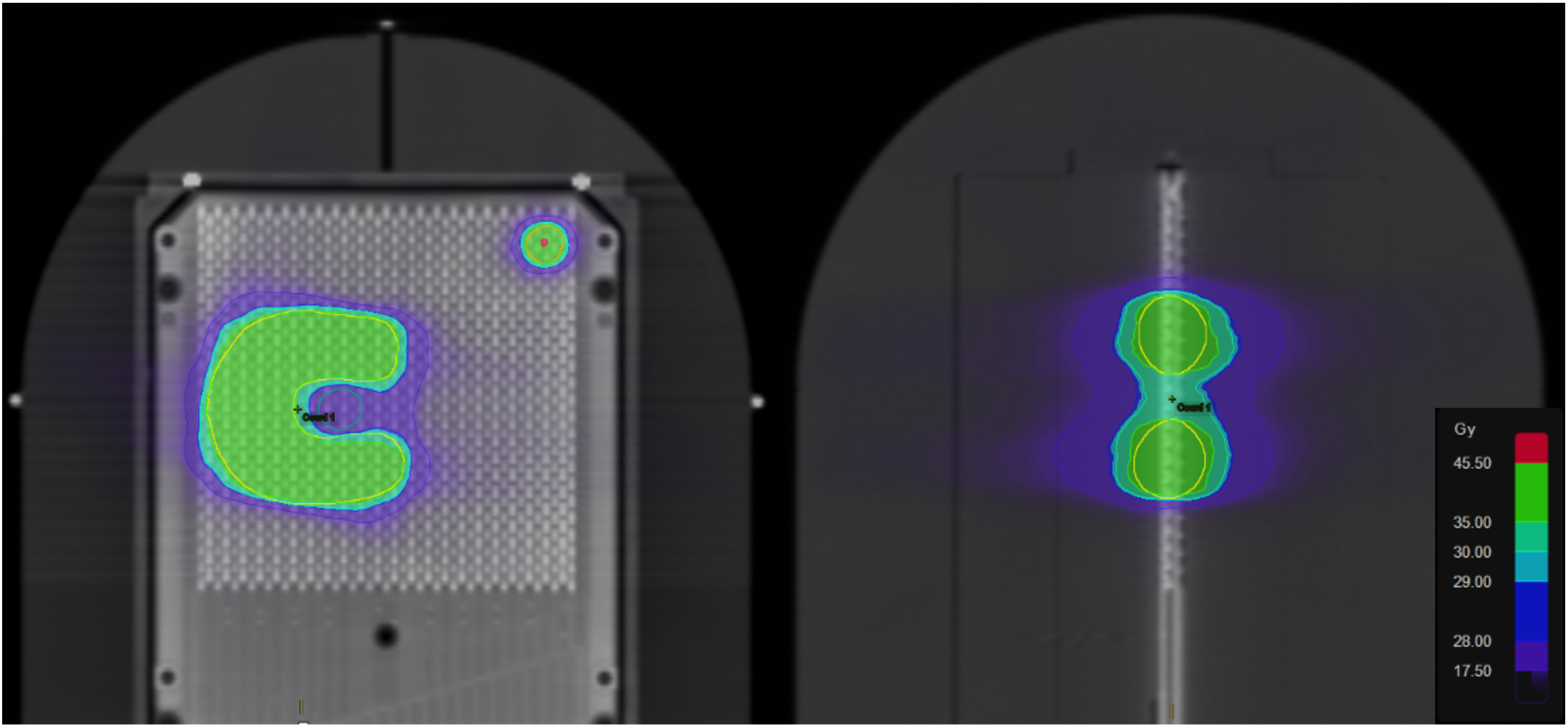


Figure S8: Coronal (left) and sagittal (right) view of case 8 targets and dose distributions. Targets are indicated by solid lines; dose is indicated by the color wash. Plan isocenter is indicated by the crosshairs.


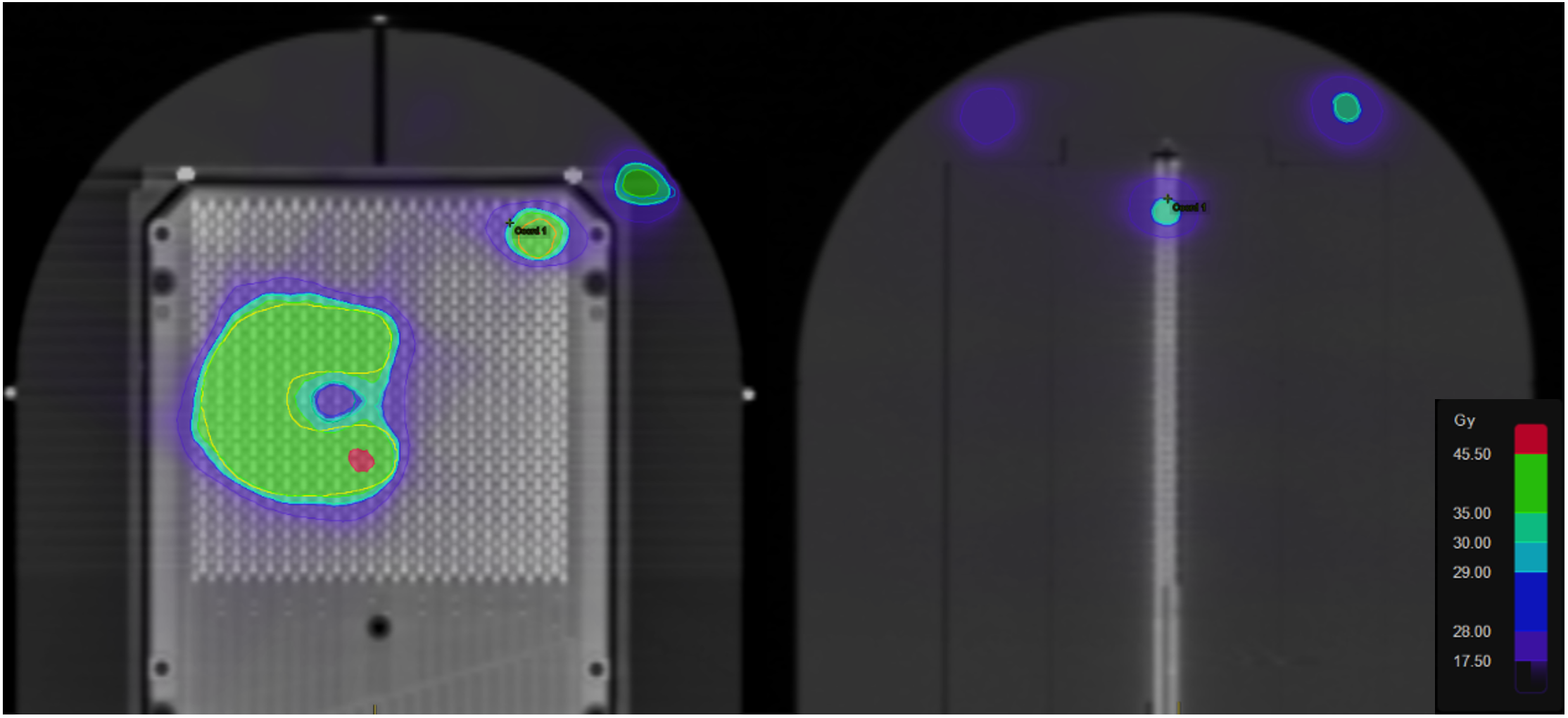


Figure S9: Coronal (left) and sagittal (right) view of case 9 targets and dose distributions. Targets are indicated by solid lines; dose is indicated by the color wash. Plan isocenter is indicated by the crosshairs. Note that targets drawn off the detector plane were used to shift the treatment isocenter.


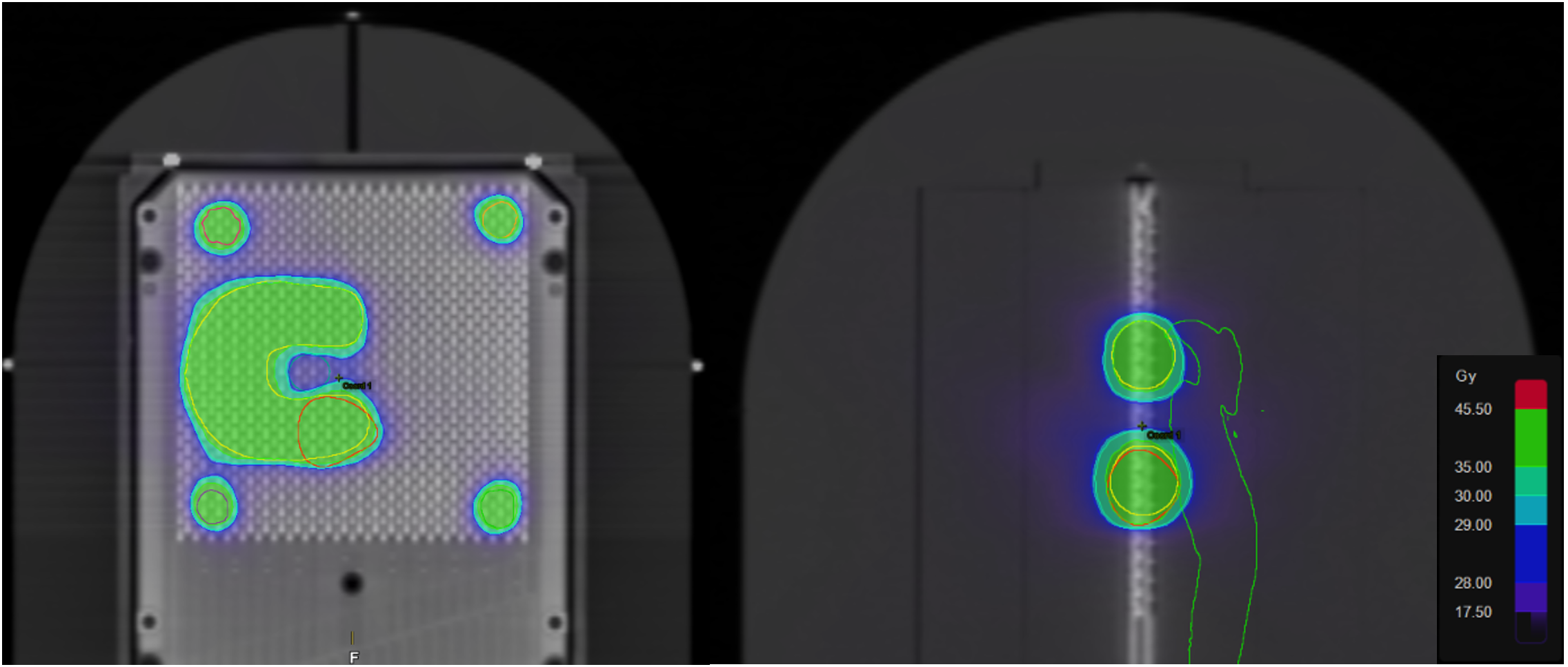


Figure S10: Coronal (left) and sagittal (right) view of case 10 targets and dose distributions. The mock brainstem was contoured in green. Targets are indicated by solid lines; dose is indicated by the color wash. Plan isocenter is indicated by the crosshairs.

**Summary of Planning Parameters:**

The summary of table angles used in treatment planning is listed in Table S1. The dose goals used for treatment plan optimization are listed in Table S2. Mock structures were contoured directly on the StereoPHAN to simulate patient geometry. We did not follow a strict V_24Gy_ constraint for the brain, since targets varied greatly in size; however, the V_24Gy_ was minimized to the greatest extent possible while meeting the target dose goals.

Table S1: Summary of table angles (IEC 61217) used for each gantry arc in test cases.

|  | **Case 1** | **Case 2** | **Case 3** | **Case 4** | **Case 5** | **Case 6** | **Case 7** | **Case 8** | **Case 9** | **Case 10** |
| --- | --- | --- | --- | --- | --- | --- | --- | --- | --- | --- |
| **Table angles [degrees]** | 0 | 0 | 0 | 0 | 0 | 0 | 10 | 0 | 0 | 0 |
|  | 30 | 0 | 0 | 0 | 0 | 30 | 10 | 0 | 0 | 0 |
|  | 80 | 20 | 40 | 30 | 40 | 30 | 50 | 20 | 20 | 0 |
|  | 300 | 20 | 40 | 30 | 40 | 60 | 50 | 20 | 20 | 40 |
|  | 330 | 60 | 80 | 90 | 80 | 60 | 50 | 60 | 20 | 40 |
|  |  | 60 | 80 | 90 | 80 | 270 | 290 | 280 | 70 | 70 |
|  |  | 290 | 300 | 300 | 300 | 300 | 290 | 280 | 70 | 70 |
|  |  | 290 | 330 | 300 | 300 | 300 | 290 | 330 | 280 | 70 |
|  |  | 330 | 330 |  | 340 |  | 320 |  | 280 | 280 |
|  |  | 330 |  |  | 340 |  | 320 |  | 280 | 280 |
|  |  |  |  |  |  |  | 350 |  | 310 | 280 |
|  |  |  |  |  |  |  | 350 |  | 310 | 310 |
|  |  |  |  |  |  |  | 350 |  | 310 | 310 |
|  |  |  |  |  |  |  |  |  |  | 340 |
|  |  |  |  |  |  |  |  |  |  | 340 |

Table *S2*: Dose goals used for planning. Mock structures were contoured on the StereoPHAN*.*

| **Structure** | **Dose goal (5 fractions)** |
| --- | --- |
| PTV | D_98.0%_ ≥ 35.0 Gy  D_min_ ≥ 32.0 Gy |
| Avoidance (C-Shape) | D_0.50cc_ ≤ 23.0 Gy  D_0.03cc_ ≤ 31.0 Gy |
| Brainstem | D_0.50cc_ ≤ 23.0 Gy  D_0.03cc_ ≤ 31.0 Gy |
| Eye | D_0.20cc_ ≤ 23.0 Gy  D_0.03cc_ ≤ 25.0 Gy |
| Optic Chiasm | D_0.20cc_ ≤ 23.0 Gy  D_0.03cc_ ≤ 25.0 Gy |
| Optic Nerve | D_0.20cc_ ≤ 23.0 Gy  D_0.03cc_ ≤ 25.0 Gy |
| Cochlea | D_0.03cc_ ≤ 25.0 Gy |
| Brain | V_24.0Gy_ minimized as reasonably achievable |

*Abbreviations: PTV, planning target volume; D_x_, dose to a volume “x”; min, minimum dose; V_y_, volume receiving at least the dose “y”.*

**Pre-Measurement Off-Isocenter Winston-Lutz Test:**

On the day of measurements, off-isocenter Winston-Lutz tests were performed. The tested combinations of gantry, couch, and collimator angles are listed in Table S3. Alignment was tested for a target at isocenter, and one 5.6 cm off-isocenter. Portal images were analyzed in RIT version 6.5.64 (Radiological Imaging Technology, Inc., Colorado Springs, CO, USA) using the 3D EPID Stereotactic Alignment module. 3-dimensional (3D) misalignments and table starshot radii calculated by the RIT program are listed in Table S4.

Table S3: Tested gantry, collimator, and table angles in the off-isocenter Winston-Lutz test performed on the day of measurements.

|  | **Gantry** | **Collimator** | **Table** |
| --- | --- | --- | --- |
| **Tested angle [degrees**] | 0 | 0 | 0 |
|  | 90 | 0 | 0 |
|  | 150 | 0 | 0 |
|  | 180 | 0 | 0 |
|  | 270 | 0 | 0 |
|  | 330 | 0 | 0 |
|  | 0 | 90 | 0 |
|  | 0 | 270 | 0 |
|  | 0 | 45 | 45 |
|  | 0 | 0 | 90 |
|  | 0 | 0 | 270 |
|  | 0 | 45 | 315 |

Table S4: Day-of-measurement results for off-isocenter Winston-Lutz test.

| **Cases measured** | **3D misalignment, isocenter [mm]** | **3D misalignment, off-iso [mm]** | **Table starshot radius, isocenter [mm]** | **Table starshot radius, off-iso [mm]** |
| --- | --- | --- | --- | --- |
| 1, 2 | 0.31 | 0.18 | 0.68 | 0.78 |
| 3, 4, 5 | 0.18 | 0.12 | 0.29 | 0.52 |
| 6, 7 | 0.20 | 0.17 | 0.39 | 0.65 |
| 8 | 0.22 | 0.15 | 0.41 | 0.70 |
| 9, 10 | 0.23 | 0.23 | 0.55 | 0.42 |
